# Multiple introductions of serotype O foot-and-mouth disease viruses into East Asia in 2010–2011

**DOI:** 10.1186/1297-9716-44-76

**Published:** 2013-09-05

**Authors:** Begoña Valdazo-González, Anna Timina, Alexey Scherbakov, Nor Faizah Abdul-Hamid, Nick J Knowles, Donald P King

**Affiliations:** 1 The Pirbright Institute, Ash Road, Pirbright, Surrey GU24 0NF, UK; 2 FGI Federal Centre for Animal Health, Vladimir, Russian Federation; 3 Department of Veterinary Services, Wisma Tani, Block Podium, Lot 4G1, Precinct 4 Federal Government Administration Centre, Putrajaya 62630, Malaysia

## Abstract

Foot-and-mouth disease virus (FMDV) is a highly contagious and genetically variable virus. Sporadic introductions of this virus into FMD-free countries may cause outbreaks with devastating consequences. In 2010 and 2011, incursions of the FMDV O/SEA/Mya-98 strain, normally restricted to countries in mainland Southeast Asia, caused extensive outbreaks across East Asia. In this study, 12 full genome FMDV sequences for representative samples collected from the People’s Republic of China (PR China) including the Hong Kong Special Administrative Region (SAR), the Republic of Korea, the Democratic People’s Republic of Korea, Japan, Mongolia and The Russian Federation were generated and compared with additional contemporary sequences from viruses within this lineage. These complete genomes were 8119 to 8193 nucleotides in length and differed at 1181 sites, sharing a nucleotide identity ≥ 91.0% and an amino acid identity ≥ 96.6%. An unexpected deletion of 70 nucleotides within the 5′-untranslated region which resulted in a shorter predicted RNA stem-loop for the S-fragment was revealed in two sequences from PR China and Hong Kong SAR and five additional related samples from the region. Statistical parsimony and Bayesian phylogenetic analysis provide evidence that these outbreaks in East Asia were generated by two independent introductions of the O/SEA/Mya-98 lineage sometime between August 2008 and March 2010. The rapid emergence of these viruses from Southeast Asia highlights the importance of adopting approaches to closely monitor the spread of this lineage that now poses a threat to livestock industries in other regions.

## Introduction

Foot-and-mouth disease (FMD) is a highly contagious viral disease characterized by rapid onset and high morbidity in a wide range of susceptible host species within the members of the order Artiodactyla (for reviews see [1,2]). The disease is endemic in the Middle East, Central and South Asia, Africa, and some countries in South America. FMD is notifiable to the World Organisation for Animal Health (OIE) and as a consequence FMD-affected countries have restricted trade in livestock and livestock products with FMD-free regions and countries. Therefore, an incursion of FMD into disease-free countries can have a devastating impact as was shown in the UK in 2001 [3] and 2007 [4], or in Taiwan during 1997 [5].

FMD is caused by a non-enveloped picornavirus (FMDV: genus *Aphthovirus*) with icosahedral symmetry. The virion, approximately 30 nm diameter, contains a single-stranded positive-sense RNA genome of approximately 8500 nucleotides (nt) in length. It contains a single open reading frame which is flanked by 5′ and 3′ untranslated regions (UTRs) and encodes the four structural proteins which form the capsid [1A (also known as VP4); 1B (VP2); 1C (VP3) and 1D (VP1)], and ten non-structural proteins (L, 2A, 2B, 2C, 3A, 3B1-3, 3C, and 3D) (for reviews see [6,7]). FMDV is a rapidly evolving virus classified into seven distinct serotypes, i.e. O, A, C, Asia 1, and Southern African Territories (SAT) 1, SAT 2 and SAT 3, which are supported by genetic classification based on the VP1-coding region. Most of our knowledge about the global distribution and molecular epidemiology of the virus is dependent upon analysis of this region which comprises approximately 8% of the FMDV genome [8]. However, complete genome sequences of FMDV are required to fully understand viral determinants of pathogenicity, virulence, host range and evolution. Moreover, complete genome sequence analysis of FMDV isolates has been successfully used to trace the origin and the transmission pathways of the virus within an outbreak [4,9-11].

During 2010–2011, incursions of the Mya-98 lineage of the Southeast Asia (SEA) topotype of serotype O (O/SEA/Mya-98) caused a series of high profile FMD outbreaks across five East Asian countries: the People’s Republic of China (PR China) including the Hong Kong Special Administrative Region (SAR), Japan, Mongolia, the Russian Federation (Russia) and the Republic of Korea (ROK; South Korea) [12-15]. A range of host species have been affected by these outbreaks including domesticated pigs, cattle and small ruminants, as well as evidence for infection in gazelles in Mongolia. Previous pandemic waves of FMD have affected many East Asian countries: during 1999–2002, the O/ME-SA/PanAsia lineage caused widespread outbreaks in PR China, ROK and Japan (in 1999–2000, 2000 and 2002 and 2000, respectively) prior to those in South Africa (2000) and Europe (2001) [3,16]. During 2005–2007, serotype Asia 1 also spread throughout many countries in the region (PR China, Mongolia, Russia, North Korea); although it was not possible to determine the precise origin of these outbreaks, Southeast Asia was not implicated [17]. In ROK and PR China the outbreaks due to O/SEA/Mya-98 were preceded by FMD outbreaks due to serotype A (A/ASIA/Sea-97 lineage) [12]. Together, these recent events may be indicative of changing epidemiology of FMD in East Asia which may heighten risk for onward transmission to more distant countries including those that are FMD-free.

The aim of this study was to analyse the complete genomes of representative FMD viruses recovered from outbreaks during the pandemic of O/SEA/Mya-98 in East Asia and to compare them with sequences from viruses from Southeast Asia where this lineage is endemic [18]. Furthermore, previously uncharacterised FMD outbreaks due to serotype O also occurred during 2011 in the Democratic People’s Republic of Korea (DRK; North Korea) and analysis of one of these samples are included in this report. This study investigates the origin and evolution of this emerging virus lineage.

## Materials and methods

### Samples

The clinical samples and isolates included in the present study are listed in Table 1. They represent viruses within the O/SEA/Mya-98 lineage which affected eight East Asian countries during 2009–2011. These isolates were selected on basis of their VP1 sequence, previously described [12].

**Table 1:** FMDV samples from the O/SEA/Mya98 lineage analysed in this study representing complete genomes (CG), S-fragments (S) and polyprotein open-reading frames (ORF) generated at the Pirbright Institute (PI) and the FGI Federal Centre for Animal Health (ARRIAH).

	**Isolate**	**Specimen**	**Location**	**Collection date**	**Host species**	**GenBank accession number (source laboratory for novel data)**
CG	O/MAY/7/2007	Cell culture	Melaka, Malaysia	20/10/2007	Cattle	HQ632772
CG	O/TAI/22/2009	Fluid/cell culture	Lamphun, Thailand	18/11/2009	Swine	KF112879: This work (PI)
CG	O/MYA/5/2009	Epithelial suspension	Bago, Myanmar	10/06/2009	Cattle	KF112880: This work (PI)
CG	O/MOG/7/2010	Epithelial suspension	Sukhbaatar Province. Mongolia	06/09/2010	Cattle	KF112881: This work (PI)
CG	O/MOG/C-10/2010	Cell culture	Mongolia	01/05/2010	Cattle	KF112882: This work (ARRIAH)
CG	O/RUS/Jul 2010	Cell culture	Abagaytuy, Zabajkal’skij Kray, Chita region, RF	05/07/2010	Swine	KF112883: This work (ARRIAH)
CG	O/RUS/Aug 2010	Cell culture	Zabajkal’skij Kray, Chita region, RF	26/08/2010	Cattle	KF112884: This work (ARRIAH)
CG	O/JPN/1/2010	Cell culture	Miyazaki Prefecture, Japan	17/04/2010	Cattle	KF112885: This work (PI)
CG	O/SKR/4/2010	Fluid/Vesicular	Ganghwa, Incheon, ROK	07/04/2010	Cattle	KF112886: This work (PI)
CG	O/SKR/5/2010	Fluid vesicular	Andong, Gyeongbuk, ROK	28/11/2010	Swine	KF112887: This work (PI)
CG	O/DRK/31/2011	Epithelial suspension	Pyongan, DPRK	01/01/2011	Cattle	KF112888: This work (PI)
CG	O/BY/CHA/2010	Epithelial suspension	Baiyun district, Guangzhou city, Guangdong, PRC	15/03/2010	Swine	JN998085
CG	O/GZ/CHA/2010	Epithelial suspension	Guanzhou, Jiangxi, PRC	15/03/2010	Cattle	JN998086
CG	O/GSLX/2010	-	Linxia, Gansu, PRC	30/06/2010	Swine	JQ900581
CG	O/CHN/Mya98/33-P	Cell culture	PRC	30/06/2010	Cattle	JQ973889
CG	O/HKN/15/2010	Epithelial suspension	Hong Kong SAR	24/02/2010	Swine	KF112889: This work (PI)
CG	O/HKN/20/2010	Epithelial suspension	Hong Kong SAR	03/03/2010	Swine	HM229661: This work (PI)
S	O/HKN/1/2010	Epithelial suspension	Hong Kong SAR	05/02/2010	Swine	KF112890: This work (PI)
S	O/HKN/4/2010	Epithelial suspension	Hong Kong SAR	10/02/2010	Swine	KF112891: This work (PI)
S	O/HKN/6/2010	Epithelial suspension	Hong Kong SAR	10/02/2010	Swine	KF112892: This work (PI)
S	O/HKN/7/2010	Epithelial suspension	Hong Kong SAR	22/02/2010	Swine	KF112893: This work (PI)
S	O/HKN/10/2010	Epithelial suspension	Hong Kong SAR	24/02/2010	Swine	KF112894: This work (PI)
S	O/HKN/12/2010	Pericardial fluid	Hong Kong SAR	24/02/2010	Swine	KF112895: This work (PI)
S	O/HKN/13/2010	Epithelial suspension	Hong Kong SAR	24/02/2010	Swine	KF112896: This work (PI)
S	O/HKN/18/2010	Epithelial suspension	Hong Kong SAR	01/03/2010	Swine	KF112897: This work (PI)
S	O/HKN/19/2010	Tissue	Hong Kong SAR	03/03/2010	Swine	KF112898: This work (PI)
ORF	O/VN/YB105/2009	Cell culture	Yen Bai, Vietnam	17/09/2009	Buffalo	GU582115
ORF	O/VN/QB88/2009	Cell culture	Quang Binh, Vietnam	09/11/2009	Cattle	GU125650
ORF	O/VN/LC169/2009	-	Lao Cai, Vietnam	15/11/2009	-	DQ119643
ORF	O/VN/GL13/2006	Cell culture	Gia Lai, Vietnam	15/04/2006	Cattle	GU125648
ORF	O/VN/SL01/2006	Cell culture	Son La, Vietnam	15/10/2006	Buffalo	GU125649
ORF	O/VN/SL21/2006	Cell culture	Son La, Vietnam	15/10/2006	Cattle	GU125647
ORF	O/VN/SL22/2006	Cell culture	Son La, Vietnam	15/10/2006	Cattle	HM055510
ORF	O/HLJOC12/2003	-	Heilongjiang, PRC	30/06/2003	-	DQ119643

### Full genome amplification

The FMDV amplification of 12 isolates was undertaken in two laboratories using the following approaches.

At The Pirbright Institute (PI, UK), the full genome of nine FMD viruses (Table 1) were amplified using a protocol initially developed to sequence related FMDV serotype O viruses from Southeast Asian countries [19]. Briefly, total RNA was extracted from the clinical samples or cell culture using RNeasy Mini Kit (Qiagen Ltd., Crawley, West Sussex, UK) according to the manufacturer’s instructions. After reverse-transcription, using the UKFMD Rev 6 primer (5′-GGC GGC CGC TTT TTT TTT TTT TTT-3′) and SuperScript™ III Reverse Transcriptase (Invitrogen, CA, USA), the viral cDNA was purified [Illustra™ GFX PCR DNA and Gel Band Purification Kit (GE Healthcare, Buckinghamshire, UK)] according to the manufacturer’s instructions, and amplified using Platinum® High Fidelity Taq (Invitrogen, CA, USA) to generate 22 PCR overlapping fragments that ranged in length from approximately 330 to 700 base pairs.

At the FGI Federal Centre for Animal Health (ARRIAH), full genome sequencing was undertaken as follows. Briefly, total RNA was extracted from three cell culture isolates (Table 1) using Ribo-prep kit (ILS, Moscow, Russian Federation) according to the manufacturer’s instructions. Reverse-transcription and PCR (variant “one tube – one buffer”) were used to generate 18 overlapping fragments. To determine 5′- and 3′-end sequences, RNA ligation was carried out using T4 RNA Ligase (Fermentas, Lithuania) in a protocol similar to one described previously [20]. Subsequently, a nested RT-PCR using forward primers for 3D region and reverse primers for the S-fragment was used to amplify a PCR-fragment consisting of approximately 155–160 nt (depending on poly(A) length) from the 3′-end and 74 nt from the S-fragment of FMDV RNA.

### Amplification of the S-fragment of the 5′-UTR

Nine additional clinical samples from animals infected within the O/SEA/Mya-98 outbreak in Hong Kong SAR (Table 1) were processed at the PI as previously described to generate the S-fragment of the 5′ UTR. The primers described in the full genome protocol to generate and sequence this region of the FMDV genome (i.e. O1F and O1R [19]), were used in parallel with a further reverse primer (i.e. O1F2: 5′-ACC GAC TAG TAC TCT TAA CAC TCC GC-3′), designed to target the deleted region within the S-fragment of the 5′ UTR. This last step was carried out to discard the hypothesis that the deletion in the S-fragment of the FMDV isolate O/HKN/20/2010 was an artefact of the RT-PCR procedure either due to the passage of the virus in cell culture.

### DNA visualization and sequencing

The PCR products were visualized on a 1.8% TBE agarose gel stained with ethidium bromide and purified using the same kit used for cDNA purification. Sequencing reactions were performed using the individual respective primers used for the amplification and Big Dye-Terminator v3.1 Cycle Sequencing Reaction Kit on an ABI 3730 DNA Analyser (Applied Biosystems, USA) following the manufacturer’s instructions. Sequences were assembled, proof-read and edited with the Lasergene version 10.1 package (DNASTAR Inc, USA). These sequences were submitted to GenBank and were assigned the following accession numbers: HM229661, KF112879-KF112898.

### Computational analysis

The sequences were compared with the complete genome sequences or sequences coding for the polyprotein of FMD viruses from the O/SEA/Mya-98 lineage obtained from other countries in East Asia such as the PR China, Malaysia and Vietnam which are available in GenBank (Table 1). Alignment of the sequences was performed using the ClustalW subroutine in BioEdit, Version 7.0.5.3 [21]. The same program was used to calculate the nucleotide and amino acid (aa) identity matrices.

Calculation of dN/dS and detection of potential positive selection was carried out using using SNAP [22]. Potential recombination was checked using SimPlot version 3.5.1 [23]. The RNA structure of the S-fragment was reconstructed for O/HKN/20/2010, O/HKN/15/2010 and O/TAI/22/2009 using RNAStructure v 3.5 [24] and RNAdraw version 1.1 [25].

Maximum-likelihood trees (1000 bootstrap replications) optimized using the heuristic nearest-neighbour-interchange method as implemented by the software program MEGA version 5.10 [26] were calculated using the S-fragment and the VP1-coding sequence. Maximum parsimony analyses using full genome sequences were carried out as implemented in TCS freeware, version 1.21 [27] following formatting using DnaSP, Version 4.10.3 [28]. The General Time Reversible (GTR) model (with no invariant sites) of nucleotide substitution was selected using jModelTest [29]. Bayesian evolutionary analysis using Markov chain Monte Carlo (MCMC) sampling bases (100 000 trees from 100 million generations), as implemented using BEAST software, version 1.6.1. [30], was carried out using the sequence coding for the polyprotein to estimate the rate of molecular evolution, to infer phylogenetic relationships and to implement the genetic temporal reconstruction of the outbreaks. Sampling collection dates were used to calibrate the molecular clock. The robustness of the parameters was assessed by substituting different combinations of molecular clocks and demographic models. The resulting output was checked in Tracer, version 1.5 [31] and visualized with FigTree, version 1.3.1. [32].

## Results

### RT-PCR and sequencing

A total of 12 full genome sequences belonging to the O/SEA/Mya-98 lineage of FMDV collected from eight Eastern Asia countries during 2009–2011 were obtained using an overlapping PCR strategy. All of the component PCR products were of the expected size and no evidence of cross-contamination was detected within the negative control RT-PCR reactions which were performed in parallel. Sequencing coverage for these consensus sequences (total number of sequenced nucleotides divided by the contig length) was > 3.5.

The novel sequences generated in this study were compared with additional FMDV sequences (five full genome sequences and eight more sequences coding for the virus polyprotein) from the same lineage available in GenBank. In total, 17 full genome sequences and 25 sequences coding for the polyprotein of FMDVs belonging to the O/SEA/Mya-98 lineage recovered from outbreaks in the PR China and Hong Kong SAR, ROK, DRK, Japan, Mongolia, Russia, Thailand, Malaysia, Myanmar and Vietnam during 2003–2011 (Table 1) were included in the following analysis.

### Analysis of the full genome sequences

The length of the 12 generated sequences ranged from 8123 to 8192 nt which for the nine sequences generated at the PI included 44–55 nt derived from the PCR primers, a 10-nt artificial internal poly(C) tract within the 5′ UTR and a 10-nt artificial poly(A) tail at the 3′ terminus of the virus. For the three genomes characterised at ARRIAH, only the poly(C) tract sequences were artificial, while the poly (A) tract was truncated to 10 nucleotides in length. Only three ambiguities in two sequences (O/JPN/1/2010 and O/MYA/5/2009) were found: R (A/G) and Y (C/T) at positions 2180 and 2182, respectively, in the first sequence; and R (A/G) at position 5792 in the second sequence.

**Figure 1:**
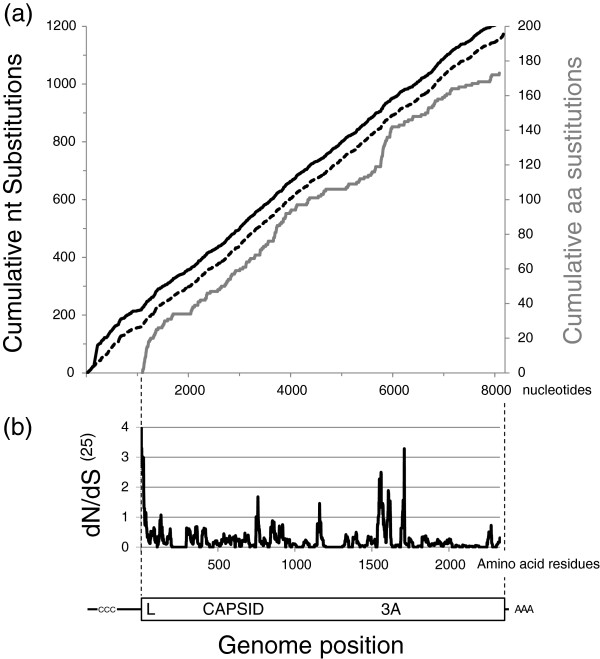
**Nucleotide and amino acid variability of the 17 O/SEA/Mya-98 FMDV genome sequences obtained from East Asia.** Graph **(a)** shows cumulative nucleotide (including [black] and excluding [black dots] the gap in the S fragment of the 5′- UTR) and amino acid (grey) substitutions against nucleotides occurring along the genome. Synonymous and nonsynonymous substitutions (mean values from a window of 25 residues sliding by an increment of one-codon) across the genome are also shown **(b)**.

The alignment of the 12 generated sequences and those available in GenBank (17 full genome sequences, 8124 sites; 8032 sites excluding sites with gaps) revealed 1186 nt substitutions at 1146 sites (Figure 1a). These substitutions were distributed across the genome (0.14 nt substitutions per nt sequenced) although rates in individual genomic regions varied from 0.25 substitutions per nt sequenced for the 3′ UTR to 0.15 (substitutions per nt sequenced) for the 5′ UTR. Within the structural proteins, VP4 had the lowest nt rate (0.10) compared to VP2 (0.13), VP1 (0.16) and VP3 (0.16). Pairwise comparisons between sequences are shown in Additional file 1. Nucleotide sequence identity ranged from 91.0% to 99.3% while predicted aa identity ranged from 96.6% to 99.5%. There was no clear evidence of recombination within these sequences or between these sequences and those available in GenBank (using SimPlot version 3.5.1; data not shown).

Within the open reading frame encoding the FMDV polyprotein, which ranged from 2331 to 2333 aa length, there were 173 aa substitutions. The difference in aa length was due to a single codon insertion in the sequences for O/GSLX/2010 and O/HKN/20/2010 after position 9 of the polyprotein and a single codon deletion in the sequences for O/SKR/4/2010 and O/CHN/Mya98/33-P at position 28 of the polyprotein. The coding region of the L protein had the highest aa substitution rate [0.17 substitutions per aa sequenced, higher (*P* < 0.05) than the most variable of the structural proteins] followed by VP1 (0.11) and 3B (0.15) as clearly visualised in the cumulative amino acid substitution plot in Figure 1a. Moreover, analysis of the synonymous versus non-synonymous ratio (dS/dN) also highlighted equivalent regions within L, VP1, 3B as well as 2C, 3A and 3C where there was evidence of positive selection (Figure 1b).

### S-fragment (5′ UTR)

**Figure 2:**
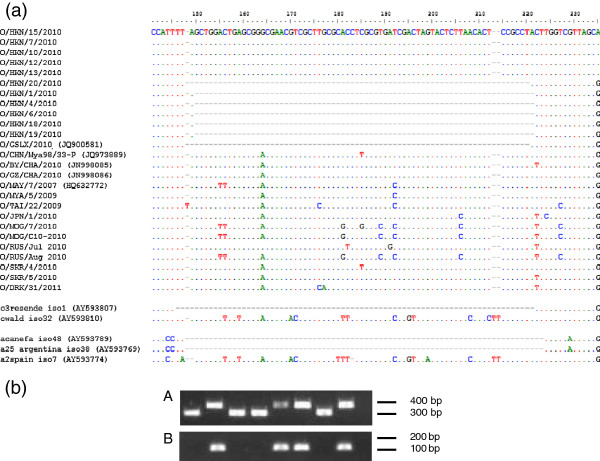
**Deletion in the S-fragment of the genome of some FMDVs belonging to the O/SEA/Mya-98 lineage. a)** Sequence alignment showing the deletion in the genome of some FMDVs belonging to the O/SEA/Mya- (70 nt length versus viruses from the same region and year) and other FMDV genomes available in GenBank. Sequences represented by names in lower case are derived from cell culture isolates generated from field strains (C_3_/Resende/BRA/1955, C/UK/149/1934, A/Canefa/ARG/1/1961; A_25_/BA/Argentina/1959 and A_2_/Spain/43); **b)** Representative results for RT-PCR amplification of the S-fragment of O/HKN/20/2010 and other related viruses from Hong Kong SAR; **A)** The use of the primer pairs O1F/O1R amplified two different products of approximately 310 and 380 bp depending on the presence or absence of the deletion, respectively. **B)** The amplification of the S-fragment of those viruses with a deletion failed when replacing the forward primer for a new primer (O1F2: 5'-ACC GAC TAG TAC TCT TAA CAC TCC GC-3′) targeting this specific region.

**Figure 3:**
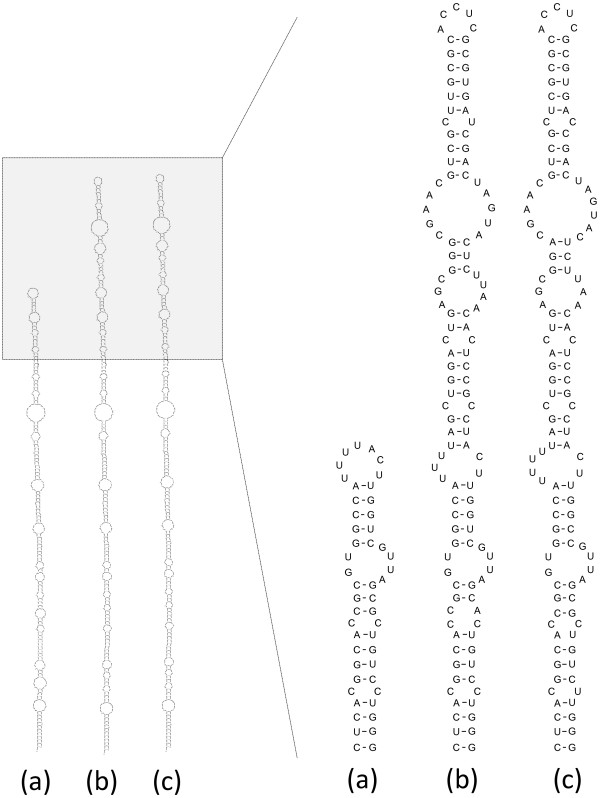
**Predicted secondary structure of the S-fragment of selected O/SEA/Mya-98 viruses.** The figure shows results corresponding to nucleotide positions 1–369 of TAI/22/2009 for **(a)** O/HKN/20/2010, **(b)** O/HKN/15/2010 and **(c)** O/TAI/22/2009.

A 70 nt deletion in the S-fragment (5′ UTR) was revealed in the sequences O/HKN/20/2010 and O/GSLX/2010 (JQ900581) which was located at positions corresponding to nt 148–217 of O/HKN/15/2010. This unexpected deletion (Figure 2a) was subsequently found in five out of nine closely related field samples from Hong Kong SAR selected by phylogenetic analysis of the VP1 region. RT-PCR results using two forward different primers (O1F and O1F2) yielded negative results when using the forward primer targeting this fragment in those viruses with the deletion (O1F2, Figure 2b) whilst positive for those viruses without the deletion, therefore discarding the hypothesis that such a deletion was an artefact of the RT-PCR procedure or due to the passage of the virus in cell culture. Moreover, identical S-fragment sequences were obtained for O/HKN/20/2010 when using epithelium suspension and cell culture as starting material. The reconstruction of the predicted secondary structure of the S-fragment for O/HKN/20/2010 showed a single stem-loop which only 35 pairs shorter in the apex than the one for O/HKN/15/2010, O/TAI/22/2009 (Figure 3).

### Maximum likelihood analysis (S-fragment and VP1 coding regions)

**Figure 4:**
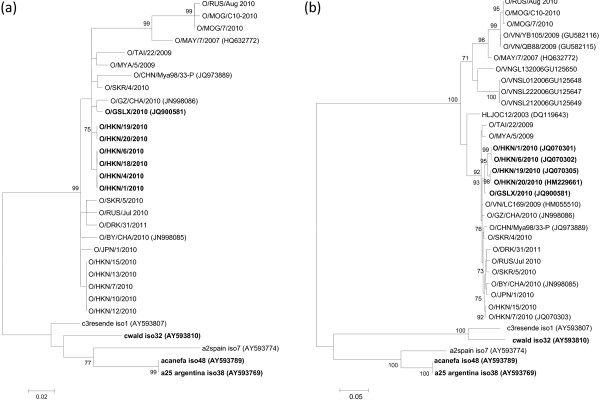
**Maximum-likelihood trees of the (a) S-fragment and (b) VP1 coding sequences of the analysed FMDVs.** The trees (1000 bootstrap replicates) were optimized using the heuristic nearest-neighbour-interchange method as implemented by the software program MEGA (version 5.10). All the sequences of the FMDV O/SEA/Mya-98 lineage present in East Asia during 2009–2011 are included, as well as other FMDV sequences including those from other serotypes in which a similar deletion in the S-fragment has been found (isolates in bold). Bootstraps values < 70% are not shown. Sequences represented by names in lower case are derived from cell culture isolates generated from field strains (C_3_/Resende/BRA/1955, C/UK/149/1934, A_2_/Spain/43), A/Canefa/ARG/1/1961 and A_25_/BA/Argentina/1959).

Phylogenetic analysis using sequences for the S-fragment of these viruses together with viruses from other FMDV serotypes in which a similar deletion is present showed that all East Asian isolates (whether they have or do not have the deletion) clustered together with other viruses within the O/SEA/Mya-98a lineage (Figure 4a) and were not closely related to other sequences from other serotypes with the deletion. Furthermore, Hong Kong SAR and Chinese viruses with the S-fragment deletion were also clustered closely together within the O/SEA/Mya-98a lineage as shown by analysis of the VP1 coding region (Figure 4b).

### Statistical parsimony analysis (17 full genome sequences)

**Figure 5:**
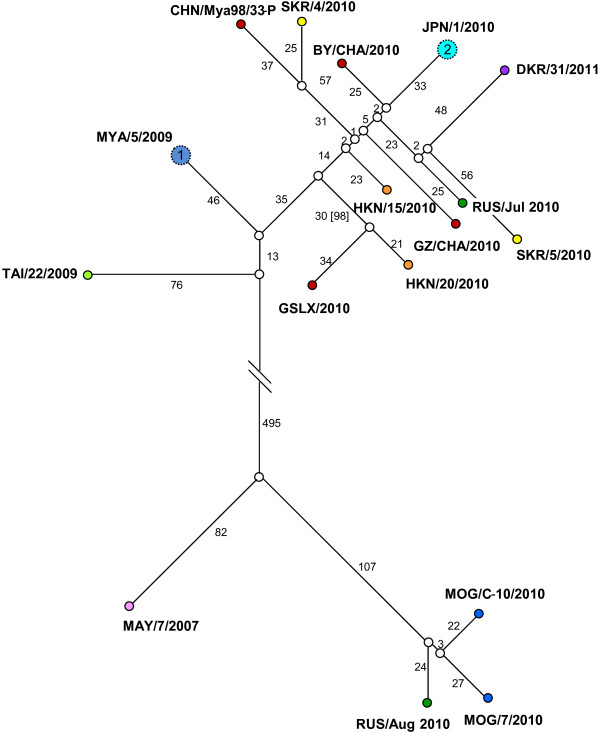
**Statistical parsimony tree as implemented by TCS using 17 full genomes of the O/SEA/Mya-98 lineage.** Representative FMD viruses causing the outbreaks in the Far East (during 2010 and 2011) are included together with three additional sequences of related viruses collected from countries (each coloured differently) where this strain is normally endemic (MAY/7/2007, MYA/5/2009 and TAI/22/2009). The number of putative nucleotide intermediates is shown for each of the branches which are drawn to scale (in brackets, number of nucleotide differences including the deletion in the 5′ UTR). Genomes that contained consensus-sequence ambiguities are shown as larger circles (labelled with the actual number of ambiguities that were present), which are linked to other genomes via the closest sequence contained within the population.

Analysis implemented by TCS allowed differences between these closely related individual sequences to be clearly visualised. Samples from the outbreaks in East Asian countries clustered into two different groups separated by more than 700 nt substitutions (Figure 5). Sequences from outbreaks in Southeast Asia, where FMDV is endemic, linked these two clusters. The groups containing these sequences did not necessarily conform to the geographical distribution of the sampling sites. The sequences from outbreaks in Thailand and Myanmar in 2009 were the closest to those causing outbreaks in the PR China and Hong Kong SAR, ROK, DRK, Japan and Russia during July 2010. The sequence from Malaysia in 2007 (O/MAY/7/2007) was the closest sequence to the viruses causing outbreaks in Mongolia and Russia during 2010.

### Markov chain Monte Carlo (MCMC) analysis (25 polyprotein sequences)

**Figure 6:**
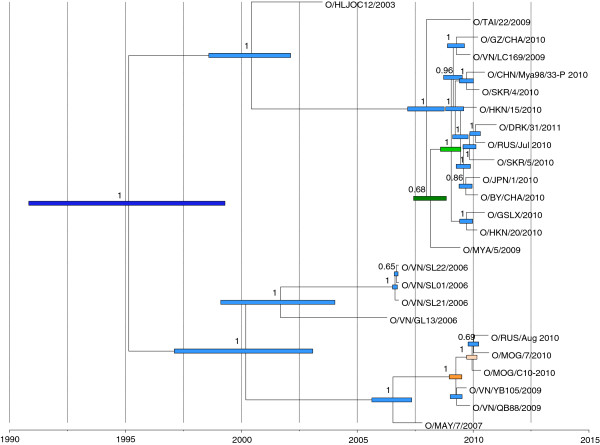
**Bayesian maximum-clade-credibility time-scaled phylogenetic tree (BEAST) using the polyprotein of 25 O/SEA/Mya-98 FMD viruses.** Uncertainty for the date of each node (95% highest posterior density - HPD - intervals) is displayed in bars (different colours for the MRCA and ancestors of different O/SEA/Mya-98 FMD sub-lineages). Only node labels with posterior probabilities > 0.6 are indicated.

A Bayesian phylogenetic tree (GTR substitution model, relaxed clock, constant population, 100 000 trees from 100 million generations) using the polyprotein of 25 O/SEA/Mya-98 FMD viruses revealed a similar topology to that generated using statistical parsimony and full genome sequences (Figure 6). The rate of nucleotide substitutions (per site per year) was 4.94 × 10^-3^ (95% highest posterior density - HPD: 3.40 × 10^-3^ - 6.58 × 10^-3^) which are comparable to the rates observed during the FMDV outbreaks in UK during 2001 and 2007 [4,9,33] and Bulgaria in 2011 [11]. The most recent common ancestor (MRCA) was estimated to be in the 1990s (21^st^ February 1995; 95% HPD: 26^th^ October 1990 -16^th^ April 1999, dark blue bar, Figure 6). The recent outbreaks occurring in the Far East during 2010 and 2011 in East Asia represented two separate clusters of the O/SEA/Mya-98 lineage (which we propose to name Mya-98a and Mya-98b). The isolates from PR China including Hong Kong SAR, ROK, DRK, Japan, and one from Russia (July 2010) and Vietnam (2009) (Mya-98a) shared a common ancestor estimated at the 19^th^ January 2009 (95% HPD: 3^rd^ August 2008 - 17^th^ July 2009; light green bar) and were derived from another ancestor common with an isolate from Myanmar (2009) estimated on the 28^th^ February 2008 (95% HPD: 6^th^ June 2007 – 3^rd^ November 2008, dark green bar). In contrast, the isolates from Mongolia and the other isolate from Russia (August 2010) (Mya-98b) were derived from a common ancestor estimated on the 13^th^ December 2009 (95% HPD: 20th September 2009 – 3rd March 2010, light orange bar) which was derived from a common ancestor shared with isolates from Vietnam 2009 estimated on the 1^st^ April 2009 (95% HPD: 20^th^ December 2008 – 5^th^ July 2009, dark orange bar).

## Discussion

Full genome sequencing data can be used to identify the origin, to reconstruct the transmission pathways and to detect undisclosed infection within field outbreaks of FMDV, as demonstrated in previous studies [4,9-11]. These data were analysed using different methods (TCS, Maximum Likelihood and Bayesian methods) providing complementary information that was used to increase the knowledge of the epidemiology of FMD in the region. The results from these different analyses were consistent and supported the occurrence of multiple independent virus introductions into some of the affected countries. This is most evident for sequences recovered from the Russian Federation, which were from two different O/SEA/Mya-98 genetic lineages. Furthermore, two sequences of FMD viruses sampled in ROK did not cluster together and were more closely related to other viruses from different countries in the region (Figure 5). The on-going risk of FMDV re-introduction influences the strategies used to control FMD. Although ROK previously held the OIE status of FMD-free without vaccination, the characterisation of these recent outbreaks has led to the use of a wide-scale vaccination programme in the country in order to adopt FMD freedom with vaccination [15]. All the viruses sequenced in this study share origins in Southeast Asia where this lineage is endemic. Certainly, the Mya-98a and Mya-98b lineages described in this paper have arisen due to at least two separate introductions into FMD-free countries in East Asia. Based upon our knowledge of previously characterised outbreaks [4,11], it is also clear that a large number of unsampled FMD cases must have occurred in order to explain the observed genetic diversity between these viruses. This data does not categorically define the number of virus introductions into East Asia, and it is now critical that further characterisation of additional samples is undertaken to further understand the epidemiology of FMD in the region to establish and reinforce control measures.

A 70 nt deletion in the S-fragment (5′ UTR) was revealed in the sequences O/HKN/20/2010 and O/GSLX/2010 (JQ900581) which was located at positions corresponding to nt 148–217 of O/HKN/15/2010. This deletion has not been reported previously for any serotype O sequence or for any FMD virus in Asia. However, previous comparative genomic studies of FMDV [7] have highlighted two instances of similar deletions for serotype A isolates from Argentina in 1959 and 1961 (AY593769 and AY593789) and one additional serotype C isolate from the UK in 1934 (AY593810). Likewise, a 41 nt deletion within the same region was found for serotype A viruses from India in 2009 (HQ832592) [34]. The results obtained in the present study using maximum likelihood analysis of the S-fragment and the VP1-coding region of isolates with and without deletions indicate that this deletion has arisen independently in these different serotypes and have not been transferred via recombination from a single event generated in one common ancestor. The deletion is neither host-species dependent, since they have been observed in viruses recovered from both pigs (East Asia) and cattle (India, South America and Europe). The reconstruction of the predicted secondary structure of the S-fragment for O/HKN/20/2010 showed a single stem-loop which was only 35 pairs shorter in the apex than the one for O/HKN/15/2010, O/TAI/22/2009 and many other FMD viruses [35,36]. Earlier studies have suggested that the S-fragment plays a role in viral replication, as well as contributing to pathogenesis [37]. However, further studies are required to determine the cause and the consequence of these changes impact upon the viral phenotype.

## Competing interests

The authors declare that they have no competing interests.

## Authors’ contributions

All authors contributed to the design of the study. BVG, NFAH, AS and AT carried out the experimental studies; BVG, NFAH, AS, NJK and DPK carried out the molecular analyses; BVG and DPK drafted the manuscript: All authors read and approved the manuscript.

## Supplementary Material

Additional file 1 **Nucleotide and amino acid identities for FMDV sequences.** Table shows percent identities for nucleotides (across the genome) and amino acids (throughout the polyprotein). Click here for file
