# Supplementary material for: Multiple introductions of serotype O foot-and-mouth disease viruses into East Asia in 2010–2011
Source: Vet Res. 2013 Sep 5;44(1):76. doi: 10.1186/1297-9716-44-76 (PMC3848691; doi:10.1186/1297-9716-44-76)
Supplement: Additional file 1 — Nucleotide and amino acid identities for FMDV sequences. Table shows percent identities for nucleotides (across the genome) and amino acids (throughout the polyprotein). [file 1297-9716-44-76-S1.docx]

**Additional file 1 Percent identities for (a) nucleotides across the genome (italics) and (b) amino acids throughout the polyprotein (top).**

| **Sequence** | **Sequence length* (nt/aa)** | **1** | **2** | **3** | **4** | **5** | **6** | **7** | **8** | **9** | **10** | **11** | **12** | **13** | **43** | **15** | **16** | **17** | **18** | **19** | **20** | **21** | **22** | **23** | **24** | **25** |
| --- | --- | --- | --- | --- | --- | --- | --- | --- | --- | --- | --- | --- | --- | --- | --- | --- | --- | --- | --- | --- | --- | --- | --- | --- | --- | --- |
| **O/MAY/7/2007 (1)** | 8111/2332 | - | 97.6 | 97.0 | 98.8 | 98.9 | 96.9 | 97.1 | 97.0 | 98.9 | 96.9 | 96.9 | 97.2 | 97.3 | 97.0 | 97.2 | 97.0 | 97.1 | 97.8 | 97.8 | 97.7 | 97.8 | 97.5 | 99.1 | 98.9 | 96.8 |
| **O/MYA/5/2009 (2)** | 8114/2332 | *92.1* | - | 98.8 | 97.2 | 97.4 | 98.7 | 98.7 | 98.7 | 97.3 | 98.7 | 98.7 | 98.9 | 99.1 | 98.8 | 98.9 | 98.7 | 98.8 | 97.0 | 97.2 | 97.1 | 97.2 | 99.3 | 97.6 | 97.4 | 97.9 |
| **O/TAI/22/2009 (3)** | 8115/2332 | *91.9* | *98.3* | - | 96.6 | 96.8 | 98.3 | 98.4 | 98.3 | 96.7 | 98.2 | 98.1 | 98.4 | 98.6 | 98.3 | 98.4 | 98.2 | 98.4 | 96.5 | 96.8 | 96.7 | 96.8 | 98.7 | 97.0 | 96.8 | 97.4 |
| **O/MOG/7/2010 (4)** | 8114/2332 | *97.3* | *91.5* | *91.4* | - | 99.4 | 96.6 | 96.8 | 96.6 | 99.3 | 96.6 | 96.5 | 97.0 | 96.9 | 96.7 | 96.9 | 96.6 | 96.8 | 97.6 | 97.5 | 97.4 | 97.5 | 97.1 | 99.4 | 99.1 | 96.4 |
| **O/MOG/C10-2010 (5)** | 8113/2332 | *97.3* | *91.5* | *91.4* | *99.3* | - | 96.7 | 96.9 | 96.8 | 99.4 | 96.7 | 96.6 | 97.0 | 97.1 | 96.8 | 96.9 | 96.8 | 96.9 | 97.7 | 97.6 | 97.5 | 97.6 | 97.2 | 99.5 | 99.3 | 96.6 |
| **O/DRK/31/2011 (6)** | 8114/2332 | *91.6* | *98.0* | *97.4* | *91.1* | *91.1* | - | 98.7 | 99.5 | 96.6 | 99.5 | 99.1 | 98.8 | 99.3 | 99.2 | 98.7 | 98.7 | 98.7 | 96.3 | 96.6 | 96.6 | 96.6 | 99.1 | 96.9 | 96.8 | 97.3 |
| **O/SKR/4/2010 (7)** | 8114/2331 | *91.9* | *98.3* | *98.0* | *91.4* | *91.4* | *98.3* | - | 98.7 | 96.8 | 98.7 | 98.8 | 98.9 | 99.2 | 98.8 | 98.9 | 98.8 | 99.3 | 96.4 | 96.7 | 96.6 | 96.7 | 99.2 | 97.1 | 97.0 | 97.4 |
| **O/SKR/5/2010 (8)** | 8116/2332 | *91.7* | *98.1* | *97.5* | *91.2* | *91.2* | *98.7* | *98.5* | - | 96.7 | 99.4 | 99.1 | 98.8 | 99.3 | 99.2 | 98.7 | 98.7 | 98.7 | 96.4 | 96.7 | 96.6 | 96.7 | 99.1 | 97.0 | 96.9 | 97.4 |
| **O/RUS/Aug 2010 (9)** | 8112/2332 | *97.3* | *91.5* | *91.4* | *99.3* | *99.3* | *91.1* | *91.3* | *91.2* | - | 96.6 | 96.6 | 96.9 | 97.0 | 96.7 | 96.9 | 96.8 | 96.8 | 97.6 | 97.5 | 97.4 | 97.5 | 97.2 | 99.4 | 99.2 | 96.5 |
| **O/RUS/Jul 2010 (10)** | 8115/2332 | *91.8* | *98.2* | *97.7* | *91.2* | *91.3* | *99.0* | *98.6* | *98.9* | *91.2* | - | 99.1 | 98.9 | 99.3 | 99.1 | 98.7 | 98.7 | 98.8 | 96.3 | 96.6 | 96.6 | 96.6 | 99.0 | 96.9 | 96.8 | 97.2 |
| **O/JPN/1/2010 (11)** | 8116/2332 | *91.9* | *98.2* | *97.7* | *91.4* | *91.4* | *98.6* | *98.7* | *98.8* | *91.4* | *99.0* | - | 98.9 | 99.3 | 99.2 | 98.8 | 98.7 | 98.8 | 96.2 | 96.6 | 96.5 | 96.6 | 99.2 | 96.9 | 96.7 | 97.2 |
| **O/HKN/20/2010 (12)** | 8048/2333 | *91.2* | *97.5* | *97.0* | *90.7* | *90.7* | *97.4* | *97.9* | *97.5* | *90.6* | *97.8* | *97.9* | - | 99.3 | 99.0 | 99.1 | 99.4 | 98.9 | 96.6 | 96.9 | 96.8 | 96.9 | 99.4 | 97.2 | 97.2 | 97.4 |
| **O/HKN/15/2010 (13)** | 8115/2332 | *91.9* | *98.5* | *98.0* | *91.4* | *91.4* | *98.7* | *99.0* | *98.8* | *91.4* | *99.0* | *99.1* | *98.0* | - | 99.5 | 99.2 | 99.2 | 99.3 | 96.6 | 96.9 | 96.8 | 96.9 | 99.5 | 97.3 | 97.2 | 97.6 |
| **O/BY/CHA/2010 (14)** | 8116/2332 | *91.8* | *98.3* | *97.8* | *91.3* | *91.4* | *98.7* | *98.9* | *98.9* | *91.3* | *99.0* | *99.2* | *97.9* | *99.2* | - | 98.9 | 98.8 | 98.9 | 96.4 | 96.7 | 96.6 | 96.7 | 99.2 | 97.0 | 96.9 | 97.4 |
| **O/GZ/CHA/2010 (15)** | 8114/2332 | *91.9* | *98.4* | *97.8* | *91.3* | *91.3* | *98.3* | *98.8* | *98.5* | *91.3* | *98.6* | *98.7* | *97.9* | *98.9* | *98.9* | - | 98.9 | 98.9 | 96.6 | 96.8 | 96.8 | 96.8 | 99.4 | 97.2 | 97.0 | 97.5 |
| **O/GSLX/2010 (16)** | 8044/2333 | *91.0* | *97.3* | *96.9* | *90.5* | *90.5* | *97.3* | *97.8* | *97.4* | *90.5* | *97.6* | *97.7* | *99.3* | *97.9* | *97.8* | *97.8* | - | 98.8 | 96.3 | 96.6 | 96.5 | 96.6 | 99.3 | 97.0 | 96.9 | 97.3 |
| **O/CHN/Mya98/33-P (17)** | 8113/2331 | *91.8* | *98.2* | *97.7* | *91.2* | *91.2* | *98.2* | *99.2* | *98.3* | *91.2* | *98.5* | *98.6* | *97.7* | *98.8* | *98.7* | *98.7* | *97.7* | - | 96.5 | 96.7 | 96.6 | 96.7 | 99.2 | 97.1 | 96.9 | 97.4 |
| **O/VN/GL13/2006 (18)** | 6996/2332 | *93.9* | *91.6* | *91.4* | *93.0* | *93.0* | *91.1* | *91.4* | *91.4* | *93.0* | *91.3* | *91.3* | *91.4* | *91.4* | *91.5* | *91.4* | *91.4* | *91.4* | - | 97.6 | 97.5 | 97.6 | 96.8 | 97.9 | 97.7 | 96.5 |
| **O/VN/SL01/2006 (19)** | 6996/2332 | *94.5* | *92.0* | *91.9* | *93.5* | *93.6* | *91.7* | *91.9* | *91.8* | *93.5* | *91.8* | *91.9* | *91.9* | *92.0* | *91.9* | *91.8* | *91.8* | *91.9* | *95.2* | - | 99.9 | 100.0 | 97.0 | 97.8 | 97.6 | 96.6 |
| **O/VN/SL21/2006 (20)** | 6996/2332 | *94.5* | *92.0* | *91.9* | *93.4* | *93.5* | *91.7* | *91.9* | *91.8* | *93.4* | *91.8* | *91.9* | *91.9* | *92.0* | *91.9* | *91.8* | *91.8* | *91.9* | *95.2* | *99.8* | - | 99.9 | 96.9 | 97.7 | 97.5 | 96.6 |
| **O/VN/SL22/2006 (21)** | 6996/2332 | *94.5* | *92.0* | *91.9* | *93.5* | *93.6* | *91.7* | *91.9* | *91.8* | *93.5* | *91.8* | *91.9* | *91.9* | *92.0* | *91.9* | *91.8* | *91.8* | *91.9* | *95.2* | *99.9* | *99.8* | - | 97.0 | 97.8 | 97.6 | 96.6 |
| **O/VN/LC169/2009 (22)** | 6996/2332 | *92.0* | *98.7* | *98.2* | *91.4* | *91.4* | *98.6* | *99.1* | *98.8* | *91.4* | *98.9* | *99.1* | *99.1* | *99.2* | *99.2* | *99.3* | *98.9* | *99.1* | *91.7* | *92.1* | *92.1* | *92.1* | - | 97.5 | 97.3 | 97.8 |
| **O/VN/QB88/2009 (23)** | 6996/2332 | *97.9* | *91.5* | *91.3* | *99.2* | *99.3* | *91.1* | *91.3* | *91.2* | *99.2* | *91.2* | *91.3* | *91.4* | *91.3* | *91.3* | *91.3* | *91.3* | *91.3* | *93.2* | *93.8* | *93.7* | *93.8* | *91.6* | - | 99.5 | 96.8 |
| **O/VN/YB105/2009 (24)** | 6996/2332 | *97.8* | *91.4* | *91.2* | *99.0* | *99.2* | *91.0* | *91.2* | *91.1* | *99.1* | *91.1* | *91.2* | *91.4* | *91.3* | *91.3* | *91.2* | *91.2* | *91.1* | *93.1* | *93.6* | *93.6* | *93.6* | *91.5* | *99.5* | - | 96.6 |
| **HLJOC12/03 (25)** | 6996/2332 | *92.1* | *95.9* | *95.6* | *91.4* | *91.5* | *95.2* | *95.6* | *95.4* | *91.4* | *95.4* | *95.5* | *95.5* | *95.7* | *95.6* | *95.6* | *95.4* | *95.5* | *92.0* | *92.3* | *92.3* | *92.3* | *95.8* | *91.6* | *91.5* | - |

* Nucleotide length excluding primers, poly(C) tract and poly(A) tail.
